# CD44v9 as a poor prognostic factor of triple-negative breast cancer treated with neoadjuvant chemotherapy

**DOI:** 10.1007/s12282-018-0888-y

**Published:** 2018-07-03

**Authors:** Eriko Tokunaga, Aya Fujita, Katsumi Takizawa, Kimiko Baba, Sayuri Akiyoshi, Yoshiaki Nakamura, Hideki Ijichi, Takanobu Masuda, Chinami Koga, Wakako Tajiri, Shinji Ohno, Kenichi Taguchi, Mayumi Ishida

**Affiliations:** 1 Department of Breast Oncology, National Hospital Organization Kyushu Cancer Center, 3-1-1 Notame, Minami-ku, Fukuoka, 811-1395 Japan; 2 Departments of Pathology, National Hospital Organization Kyushu Cancer Center, 3-1-1 Notame, Minami-ku, Fukuoka, 811-1395 Japan; 3 Breast Cancer Center, Cancer Institute Hospital, 3-8-31 Ariake, Koutou-ku, Tokyo, 135-8550 Japan

**Keywords:** Triple-negative breast cancer, Neoadjuvant chemotherapy, CD44v9, Cancer stemness

## Abstract

**Background:**

Neoadjuvant chemotherapy (NAC) is the standard therapeutic strategy for triple-negative breast cancer (TNBC). TNBC patients with residual disease after NAC have a significantly worse survival than those with pathological complete response (pCR); however, there is no apparent prognostic factor for non-pCR patients. Cancer stemness or epithelial–mesenchymal transition (EMT) might influence the sensitivity to chemotherapy.

**Patients and methods:**

Forty-eight patients with TNBC who were treated with NAC were available were included in this study. The expressions of stemness marker CD44v9, EMT marker vimentin and BRCA1, and basal phenotype were evaluated with immunohistochemistry. The relationships between the expression of these proteins and the pCR rate and the prognosis, especially in the patients with residual tumors, were investigated.

**Results:**

Among the 48 patients, pCR was achieved in 14 cases. High nuclear grade and basal phenotype in the pre-NAC samples were significantly correlated with pCR (*p* = 0.0458 and 0.0343). There were no significant relationships between the pCR rate and the expression of CD44v9, vimentin, or BRCA1. Achieving pCR was significantly correlated with longer distant metastasis-free survival (DMFS) (*p* = 0.0206). High CD44v9 expression was significantly associated with shorter DMFS (*p* = 0.0291). Among the patients in whom pCR was not achieved, high grade in the residual tumor cells, poor pathological response and high CD44v9 expression in the pre-treatment CNB samples were significantly correlated with a poor DMFS (*p* = 0.0433, 0.0406 and *p* = 0.0333). In addition, high grade in the residual tumor cells was significantly associated with high CD44v9 expression in the pre-treatment CNB (*p* = 0.0389).

**Conclusions:**

High CD44v9 expression in pre-NAC samples was associated with poor prognosis in TNBC patients treated with NAC, especially for those in whom pCR was not achieved.

**Electronic supplementary material:**

The online version of this article (10.1007/s12282-018-0888-y) contains supplementary material, which is available to authorized users.

## Introduction

Triple-negative breast cancer (TNBC), which accounts for about 15% of breast cancer cases, is defined as estrogen receptor (ER)-negative, progesterone receptor (PgR)-negative, and human epidermal receptor 2 (HER2)-negative disease. TNBC generally has a high-grade and aggressive phenotype with early distant metastasis after primary treatment, and the prognosis of patients with TNBC is often poor. Because of the lack of effective therapeutic targets, conventional chemotherapy is still considered the standard treatment for TNBC [1].

Neoadjuvant chemotherapy (NAC) is a standard therapeutic strategy for TNBC. Despite their overall poor survival, a subset of TNBC patients responds well to the standard chemotherapy. The prognosis of the patients in whom pathological complete response (pCR) is achieved is quite favorable, similar to that of other breast cancer subtypes. However, TNBC patients with residual disease after NAC have significantly worse survival than those who achieve pCR [2, 3]. Therefore, it is meaningful to investigate the factors influencing the efficacy of NAC and the prognosis after NAC, especially for patients who fail to achieve pCR.

Recent studies have shown that TNBC is a highly heterogeneous disease and is classified into several subgroups on the basis of different approaches, classical pathology, mRNA expression profile, and DNA sequencing, including analyses of copy number variations and structural rearrangements and other molecular methods [4–7]. Phenotypes related to the epithelial–mesenchymal transition (EMT) and cancer stemness are also characteristic of TNBC [8]. There are several predictive factors that influence the sensitivity to NAC in TNBC, including high nuclear grade, high proliferation rate, high immune cell infiltration, alteration of DNA repair-related genes, EMT-related gene expression, and cancer stemness [9–12]. Among the molecular subtypes of TNBC, the basal-like 1(BL1) subtype is associated with higher pCR rate than others [5, 6, 13].

Among DNA repair-related genes, low expression of BRCA1 or “BRCAness” features is correlated with taxane resistance [14–16] and a poor prognosis, specifically TNBC [17, 18]. Regarding EMT, we previously reported that vimentin, the major EMT-related factors, is poor prognostic factors for TNBC [19, 20]. However, the relationship between the expression of vimentin and the pathological response to NAC is not clear.

The adhesion molecule CD44 is expressed in cancer stem-like cells (CSCs). CD44 variant 9 (CD44v9), a splicing variant of CD44, has emerged as a novel marker of cancer stemness in a variety of solid tumors [21–23]. CSCs with high expression of CD44v have an enhanced capacity for GSH synthesis and defense against reactive oxygen species, resulting in resistance to various therapeutic stresses [23]. CD44v9 has been studied in various malignant tumors, including gastric cancers and head and neck squamous cell carcinomas (HNSCC) and urothelial cancer [21, 22, 24]. The high expression of CD44v9 has been shown to be associated with resistance to pre-surgical treatment in HNSCC [21]. However, there have been no reports regarding the clinical significance and the association with chemosensitivity of CD44v9 in breast cancer.

In the present study, we first would like to investigate the relationships between the expression of CD44v9 in pre-NAC tumor samples and the pathological response to NAC in TNBC. In addition, we evaluated the expression of BRCA1, vimentin, and basal phenotype, which are considered to be related to the chemosensitivity or prognosis of TNBC. The factors to be related to the prognosis of the patients, who fail to achieve pCR, were also examined.

## Patients and methods

### Patient population

A total of 465 patients with clinical Stage I–III primary breast cancer were treated with NAC in the Department of Breast Oncology, National Hospital Organization Kyushu Cancer Center, between 2002 and 2017. Chemotherapy was administered according to the standard regimen of anthracyclines and/or taxanes. Among 419 patients for whom the data of ER, PgR, and HER2 were available, 73 had TNBC. Both the samples from a core needle biopsy (CNB) before NAC and those obtained during surgery were available in only 48 patients. These 48 cases were thus included in this study. The clinical data were obtained from the patients’ medical records. The AJCC/UICC TNM classification and stage groupings were used. For tumor grading, nuclear grade was determined as previously described [25], because in some CNB samples the evaluation of the tubular formation was difficult due to small number of the tumor cells. Written informed consent was obtained from all of the patients before collecting the tissue samples. This study was approved by the institutional review board of our hospital.

The expression of ER and PgR was regarded as positive if the nuclear expression was ≥ 1%. The HER2 status was evaluated according to the recommendation of ASCO/CAP. The pathological responses of the tumor and dissected lymph nodes were classified based on the evaluation criteria of the Japanese Breast Cancer Society [26]; Grade 0, no response or hardly any changes in cancer cells following treatment; grade 1a, mild response recognized as mild cancer cell changes regardless of the site, or marked cancer cell changes in < 1/3 of the total number of cancer cells; grade 1b, moderate response as shown by marked changes in ≥ 1/3 but < 2/3 of the total number of cancer cells; grade 2, marked response or marked changes in ≥ 2/3 of the total number of cancer cells; and grade 3, lack of residual cancer cells, necrosis or disappearance of all cancer cells, or replacement of all cancer cells by granuloma-like and/or fibrous tissue. The pathological complete response (pCR) was regarded as the total disappearance of infiltrates, including lymph node infiltrates, regardless of the presence of residual ductal carcinoma in situ. Among the 48 patients, pCR was achieved in 14 (29.2%). The background characteristics are shown in Table 1.

**Table 1:** Background of the patients

Factors	*n* (%)
Age (years old)	
Mean (range)	51.5 (27–66)
Clinical stage	
I	2 (4.2)
IIA	17 (35.4)
IIB	10 (20.8)
IIIA	8 (16.7)
IIIB	4 (8.3)
IIIC	7 (14.6)
Menopausal status	
Premenopausal	25 (52.1)
Postmenopausal	22 (45.8)
Unknown	1 (2.1)
Tumor grade	
1	8 (16.7)
2	9 (18.7)
3	31 (64.6)
Pathological efficacy	
Non-pCR	34 (70.8)
pCR	14 (29.2)

### Immunohistochemistry

The expression of CD44v9, BRCA1, vimentin, CK5/6, and EGFR was analyzed with immunohistochemistry (IHC) in the pre-NAC samples obtained by core needle biopsy. In addition, the CD44v9 expression in the residual tumors after NAC was evaluated. The primary antibodies used were as follows: CD44v9 (RV3, 1:2500; Cosmo Bio, Tokyo, Japan), BRCA1 (MS110, 1:150; Calbiochem, Darmstadt, Germany), vimentin (V9, 1:500; Dako, Glostup, Demark), CK5/6 (D5/16 B14, 1:250; Dako), and EGFR (6B6, 1:50; Cell Signaling, Danvers, MA, USA). CD44v9 was expressed in the membrane and the cytoplasm. The expression was scored with the proportion score (0, none; 1, < 1/100; 2, 1/100–1/10; 3, 1/10–1/3; 4, 1/3–2/3; and 5, > 2/3) and intensity score (0, none; 1, weak, 2, intermediate; and 3, strong). The intensity scores of most samples were 2 and 3, and the standard cutoff has not been established for CD44v9; we therefore regarded the intensity as high if the proportion score of membranous and/or cytoplasmic expression was ≥ 4. Any distinct positive staining of the tumor cytoplasm in cancer cells with the vimentin antibody was regarded as positive vimentin expression [20]. The expression of CK5/6 was defined as positive when cytoplasmic and/or membranous staining was observed and otherwise was considered negative [20]. The expression of BRCA1 was regarded as positive when nuclear staining was observed in ≥ 10% of the cancer cells [14]. TNBC with positive expression of CK5/6 and/or EGFR was defined as basal-like breast cancer (BLBC; basal phenotype) [20].

### Statistical analyses

The statistical analyses were performed using the JMP software package, version 9.0.2 (SAS Institute Inc., Cary, NC, USA). The associations between the clinicopathological characteristics were assessed using *χ*^2^ tests. The distant metastasis-free survival (DMFS) was defined as the time from surgery to the first distant breast cancer event. Survival curves were plotted using the Kaplan–Meier method, and the association between the survival and each variable was determined by the log-rank test. Differences were considered to be significant at *p* < 0.05.

## Results

### Expression of vimentin, BRCA1, and CD44v9 and basal phenotype

**Fig. 1:**
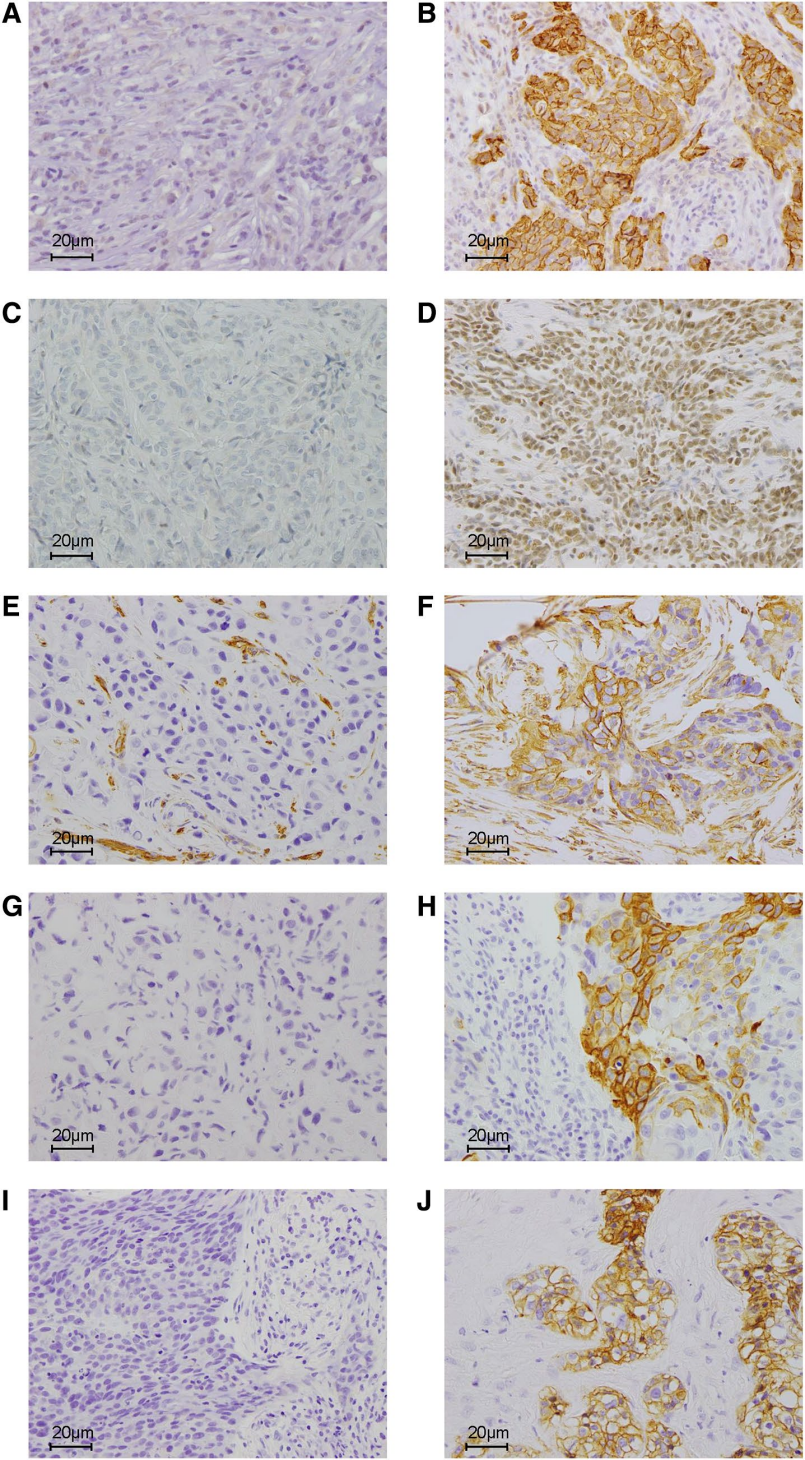
Representative pictures of the negative and positive expression of each protein evaluated by immunohistochemistry. **a** CD44v9 negative. **b** CD44v9 positive. **c** BRCA1 negative. **d** BRCA1 positive. **e** Vimentin negative. **f** Vimentin positive. **g** CK5/6 negative. **h** CK5/6 positive. **i** EGFR negative. **j** EGFR positive

The expression of CD44v9, vimentin, BRCA1, CK5/6, and EGFR was evaluated with IHC. Regarding CD44v9, 30 tumors (62.5%) were judged as having high expression, and 18 (37.5%) were regarded as having low expression. The expression of vimentin and BRCA1 was positive in 26 (54.2%) and 38 cases (79.2%). CK5/6 and EGFR were positive in 34 (70.8%) and 33 (68.5%) tumors, and 42 cases (87.5%) were regarded as basal phenotype. Representative pictures of negative and positive expression of each protein are shown in Fig. 1. The vimentin expression was positively correlated with the basal phenotype (*p* = 0.0013); however, there were no significant associations among basal phenotype, BRCA1, and CD44v9 (Table S1).

### Relationships between the nuclear grade, each protein expression, and the pathological efficacy of NAC

Table 2 shows the relationships between the nuclear grade (NG), each protein expression in the pre-treatment CNB samples, and the pathological response. A higher NG and basal phenotype were significantly correlated with a pCR (*p* = 0.0458 and 0.0343), although there was no significant relationship between the basal phenotype and NG. There were no significant relationships between the expression of vimentin, BRCA1, and CD44v9, and the pCR rate (Table 2). In addition, there were no significant relationships between the BRCA1 expression and the sensitivity to taxanes in this cohort (data not shown).

**Table 2:** Relationships between nuclear grade, protein expression, and pathological efficacy

Factors	Pathological efficacy		*p* value
	non-pCR (*n* = 34)	pCR (*n* = 14)	
Tumor grade			
1 (*n* = 8, 16.7%)	8 (23.5)	0 (0)	0.0458
2 (*n* = 9, 18.9%)	6 (17.7)	3 (21.4)	
3 (*n* = 31, 64.6%)	20 (58.8)	11 (78.6)	
Basal phenotype			
Negative (*n* = 14, 29.2%)	6 (17.7)	0 (0)	0.0343
Positive (*n* = 34, 70.8%)	28 (82.3)	14 (100)	
Vimentin			
Negative (*n* = 22, 45.8%)	18 (52.9)	4 (28.6)	0.1182
Positive (*n* = 26, 54.2%)	16 (47.1)	10 (71.4)	
BRCA1			
Negative (*n* = 10, 20.8%)	9 (26.5)	1 (7.1)	0.1053
Positive (*n* = 38, 79.2%)	25 (73.5)	13 (92.9)	
CD44v9			
Low (*n* = 18, 37.5%)	12 (35.3)	6 (42.9)	0.6244
High (*n* = 30, 62.5%)	22 (64.7)	8 (57.1)	

### Relationships between the clinicopathological factors, each protein expression, and the prognosis

**Fig. 2:**
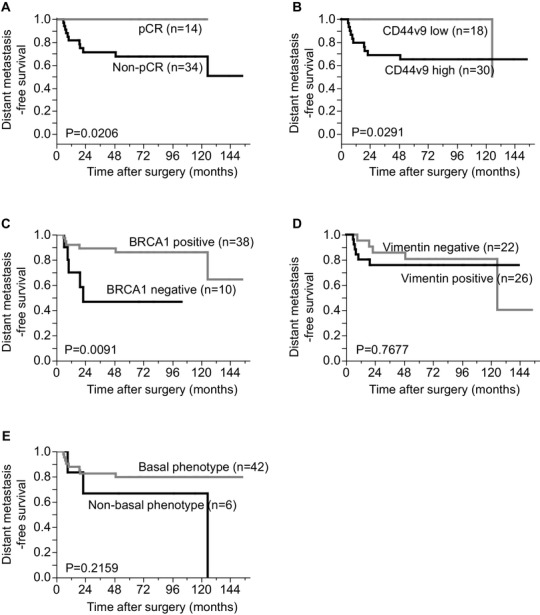
Relationships between the pCR and the expression of each protein and the distant metastasis-free survival. **a** pCR vs. non-pCR. **b** Low CD44v9 expression vs. high CD44v9 expression. **c** Negative BRCA1 vs. positive BRCA1. **d** Negative vimentin vs. positive vimentin. **e** Basal vs. non-basal phenotype

Then, the relationships between the clinicopathological factors, each protein expression, and the prognosis were examined. Median follow-up time period was 72.5 (5.7–153.8) months. As shown in Fig. 2, non-pCR, a high CD44v9 expression, and negative BRCA1 expression were significantly associated with a shorter DMFS (*p* = 0.0206, *p* = 0.0291 and 0.0091, Fig. 2a–c). There were no significant relationships between the expression of vimentin or basal phenotype and the prognosis (Fig. 2d, e). In addition, the NG of the tumor cells in the pre-treatment CNB samples was not related to the prognosis (data not shown).

### The prognosis of the patients in whom apCR was not achieved

**Fig. 3:**
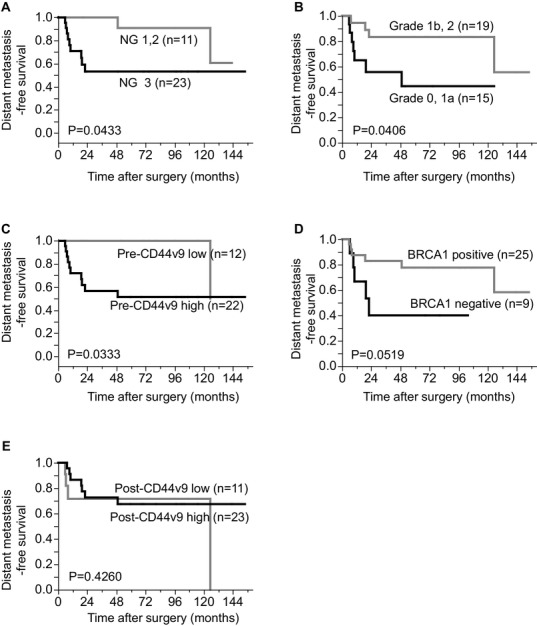
Relationships between the nuclear grade and the expression of each protein in the residual tumor cells and the distant metastasis-free survival. **a** Nuclear grade (NG) 1 or 2 vs. 3 of the residual tumor cells. **b** The pathological responses of the tumor; Grade 0, 1a vs. 1b, 2. **c** Low CD44v9 expression vs. high CD44v9 expression in the pre-NAC tumors. **d** Negative BRCA1 vs. positive BRCA1 in the pre-NAC tumors. **e** Low CD44v9 expression vs. high CD44v9 expression in the post-NAC tumors

For the patients in whom pCR was not achieved, high NG in the residual tumor cells and poor therapeutic response (Grade 0 and 1a) were significantly correlated with poorer DMFS (*p* = 0.0433 and 0.0406, Fig. 3a, b). High CD44v9 expression in the pre-treatment CNB samples was also significantly correlated with a shorter DMFS in these patients (*p* = 0.0333, Fig. 3c). The DMFS of the non-pCR patients with negative BRCA1 was shorter than that of the patients with positive BRCA1, although not statistically significant (Fig. 3d). In these 34 cases without pCR, high CD44v9 expression was observed in 23 (67.6%) cases. There was no difference in DMFS among the patients with low and high CD44v9 expression in the residual tumor cells (Fig. 3e).

Univariate and multivariate analyses were performed to assess the effects of the clinicopathological factors on DMFS in the non-pCR cases (Table 3). According to the univariate analysis, high pre-NAC CD44v9 expression was significantly associated with poor DMFS in addition to the pathological lower therapeutic response (grade 0, 1a), pathological positive lymph node metastasis and post-NAC high NG. However, a multivariate Cox hazard analyses showed that high pre-NAC CD44v9 expression was not an independent prognostic factor. On the other hand, the pathological lower therapeutic grade, pathological lymph node metastasis, and post-NAC high NG were independent poor prognostic factors in terms of DMFS.

**Table 3:** Univariate and multivariate analysis for distant metastasis-free survival in the non-pCR cases

Factors	Univariate analysis			Multivariate analysis		
	HR	95% CI	*p* value	HR	95% CI	*p* value
Pre-NAC nuclear grade						
3 vs. 1, 2	1.55	0.46–5.95	0.4753			
Pre-NAC basal phenotype						
Positive vs. negative	0.70	0.20–3.19	0.6056			
Pre-NAC vimentin						
Positive vs. negative	1.86	0.55–6.52	0.3085			
Pre-NAC BRCA1						
Positive vs. negative	0.31	0.09–1.12	0.0730			
Pre-NAC CD44v9						
high vs. low	6.86	1.31–126.1	0.0189	2.23	0.22–55.8	0.5196
Pathological therapeutic grade						
0, 1a vs. 1b, 2	3.41	1.01–13.2	0.0474	10.50	2.22–78.8	0.0023
pLN meta.						
Positive vs. negative	1.72	1.70–43.9	0.0051	25.90	4.38–277.5	0.0001
Post-NAC nuclear grade						
3 vs. 1, 2	4.44	1.10–29.9	0.0346	13.00	2.02–160.3	0.0046
Post-NAC CD44v9						
High vs. low	0.61	0.18–2.33	0.4427			

*pLN meta*. pathological lymph node metastasis

### Relationships between the NG in the residual tumors and the CD44v9 expression

**Fig. 4:**
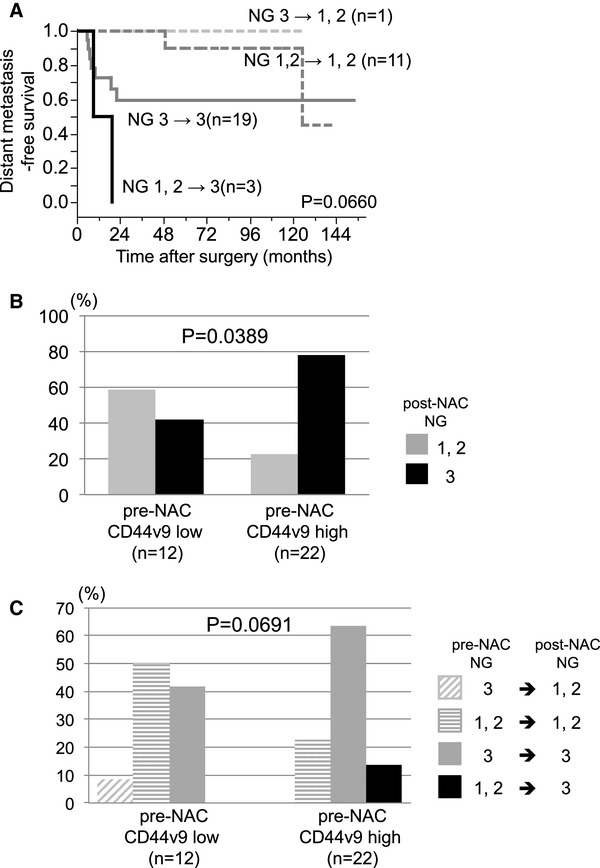
Relationships between the changes of the tumor grade and the prognosis according to the CD44v9 expression prior to NAC. **a** Distant metastasis-free survival according to the changes of the nuclear grade (NG). **b** Relationship between the NG of the residual tumor cells and the expression of CD44v9 in the pre-NAC tumor cells. **c** Relationship between changes in the NG of the tumor cells and the expression of CD44v9 in the pre-NAC tumor cells

Because the NG in the residual tumors was significantly correlated with the DMFS in the non-pCR patients, the relationships between the changes in NG and the DMFS were investigated. The prognosis of the patients with down-grade (from NG3 to NG 1, 2) or remaining NG 1, 2 was better than that of the patients with remaining NG3. In addition, two patients in whom the NG increased from 1 to 2 to 3 after NAC experienced extremely early distant recurrence (Fig. 4a). As shown in Fig. 4b, the expression of CD44v9 in the pre-NAC tumor samples was significantly correlated with the NG of the residual tumor cells. Both of the two patients in whom the NG increased from 1 to 2 to 3 after NAC had high pre-NAC CD44v9 expression and only one patient in whom the NG decreased from 3 to 1, 2 after NAC had low pre-NAC CD44v9 expression (Fig. 4c). Thus, pre-NAC CD44v9 expression was not an independent prognostic factor in non-pCR cases, partially due to the significant association between pre-NAC CD44v9 expression and NG of the residual cells. On the other hand, there are no significant association between the expression of CD44v9 and NG in the pre-NAC samples; NG1/2/3 16.7/16.7/66.7% in CD44v9-low cases (*n* = 18), NG1/2/3 16.7/20.0/63.3% in CD44v9-high cases (*n* = 38) (*p* = 0.957).

## Discussion

In this study, we investigated the clinical significance of the expressions of CD44v9, a protein related to the cancer stemness in TNBC patients who received NAC. We showed that high CD44v9 expression was significantly correlated with poor prognosis, especially in patients who failed to achieve a pCR. The NG of the residual tumor cells was significantly associated with shorter DMFS, and high CD44v9 expression prior to NAC was significantly associated with the high NG in the residual tumors. To our knowledge, this is the first report of the relationship between the CD44v9 expression and the pathological response to NAC and the prognosis in TNBC.

The major prognostic factors after NAC are pathological response (pCR or non-pCR), the residual cancer burden (RCB), and tumor proliferation, such as mitotic counts and the Ki67 index, in the residual tumor cells [25, 27–29]. The significantly poor prognosis of the patients in whom pCR was not achieved by NAC is an urgent issue to be overcome, especially for TNBC. One possible strategy for resolving this issue is the addition of further treatment after NAC and surgery. The recent Create-X study revealed the effectiveness of the adjuvant capecitabine for non-pCR patients. The disease free and overall survival were significantly longer in the capecitabine group than in the control group, especially in TNBC patients [30].

Not only the efficacy of NAC but also the prognosis after NAC is extremely different among the TNBC subtypes [5, 6, 31]. The basal-like 1 (BL1) subtype has been reported to be highly sensitive to NAC in TNBC. In the present study, the basal phenotype determined by IHC was significantly associated with high pCR rate, although it is difficult to distinguish between BL1 and BL2 by IHC alone.

Based on the findings of previous studies regarding CD44v9, we expected the pre-NAC expression of CD44v9 to be related to the subsequent efficacy of NAC [21–23]. However, the CD44v9 expression in pre-NAC tumors was not related to the pathological response on the whole. Nevertheless, high CD44v9 expression was significantly correlated with poor prognosis, especially in the patients in whom pCR had not been achieved. Multivariate analysis could not reveal that high CD44v9 in pre-NAC samples was independent prognostic factor in the non-pCR patients. Intriguingly, high CD44v9 expression in pre-NAC tumors was significantly correlated with high NG in the residual tumor cells after NAC. This seems to have contributed to the poor prognosis.

In contrast, low CD44v9 expression before NAC was correlated with better prognosis, even in the non-pCR cases. As mentioned above, the Create-X study showed that the addition of capecitabine after surgery improved the prognosis of non-pCR HER2-negative breast cancer after NAC. Thus, high CD44v9 expression may be useful marker suggesting the need for additional treatment for patients with non-pCR tumors after NAC. Sulfasalazine (SSZ), which is used for rheumatoid arthritis (RA), inhibits glutamate–cysteine transport and has been reported to suppress the CD44v-dependent tumor growth and increase the sensitivity to cytotoxic drugs in urogenital cancer [32]. A clinical study to confirm the utility of SSZ has already been started for several malignancies, including gastric cancer [33]. Reducing the CD44v9 expression or CD44v-dependent tumor growth is expected to improve the sensitivity to chemotherapy and the prognosis. In this study, there was no difference in DMFS and the NG of the residual tumor cells among the patients with low and high CD44v9 expression in the residual tumor cells. The reason of this phenomenon is not clear. The mechanism of the regulation of the expression of CD44v9 is not fully elucidated. The manipulation by the chemotherapy might modulate the expression of CD44v9 irrespective of the sensitivity to NAC.

The loss of BRCA1 expression was observed in about 20% of patients and was correlated with a poor prognosis in this study. These findings are consistent with those of previous reports [17, 18], although no association was noted between the resistance to taxanes and the loss of BRCA1 expression in this study. The EMT phenotype is considered to be related to chemoresistance. However, in the present study, there is no statistical difference in the sensitivity to NAC between vimentin-negative or positive tumors. This is probably because EMT is not defined by the expression of vimentin alone and there are many factors that determine the chemosensitivity other than EMT phenotype. Thus, in the present study, it was difficult to show the relationship between EMT evaluated based on the expression of vimentin and chemoresistance.

Several limitations associated with the present study warrant mention. The number of patients included in this study was very small, and the analysis was retrospective. To confirm the findings obtained in this study, analyses in a larger cohort are necessary.

In conclusion, the CD44v9 expression in pre-NAC tumors predicts poor prognosis after NAC in TNBC. The combined treatment of NAC and therapy targeting CD44v9 may be useful in future therapeutic regimens.

## Electronic supplementary material

Below is the link to the electronic supplementary material.

Supplementary material 1 (XLSX 36 KB)

## References

[1] Cleator S, Heller W, Coombes RC (2007). Triple-negative breast cancer: therapeutic options. Lancet Oncol.

[2] Carey LA, Dees EC, Sawyer L, Gatti L, Moore DT, Collichio F (2007). The triple negative paradox: primary tumor chemosensitivity of breast cancer subtypes. Clin Cancer Res.

[3] Liedtke C, Mazouni C, Hess KR, Andre F, Tordai A, Mejia JA (2008). Response to neoadjuvant therapy and long-term survival in patients with triple-negative breast cancer. J Clin Oncol.

[4] Burstein MD, Tsimelzon A, Poage GM, Covington KR, Contreras A, Fuqua SA (2015). Comprehensive genomic analysis identifies novel subtypes and targets of triple-negative breast cancer. Clin Cancer Res.

[5] Lehmann BD, Bauer JA, Chen X, Sanders ME, Chakravarthy AB, Shyr Y (2011). Identification of human triple-negative breast cancer subtypes and preclinical models for selection of targeted therapies. J Clin Invest.

[6] Lehmann BD, Jovanovic B, Chen X, Estrada MV, Johnson KN, Shyr Y (2016). Refinement of triple-negative breast cancer molecular subtypes: implications for neoadjuvant chemotherapy selection. PLoS One.

[7] Denkert C, Liedtke C, Tutt A, von Minckwitz G (2017). Molecular alterations in triple-negative breast cancer-the road to new treatment strategies. Lancet.

[8] Prat A, Perou CM (2011). Deconstructing the molecular portraits of breast cancer. Mol Oncol.

[9] Visvader JE, Lindeman GJ (2012). Cancer stem cells: current status and evolving complexities. Cell Stem Cell.

[10] Fleisher B, Clarke C, Ait-Oudhia S (2016). Current advances in biomarkers for targeted therapy in triple-negative breast cancer. Breast Cancer (Dove Med Press).

[11] Nakai K, Mitomi H, Alkam Y, Arakawa A, Yao T, Tokuda E (2012). Predictive value of MGMT, hMLH1, hMSH2 and BRCA1 protein expression for pathological complete response to neoadjuvant chemotherapy in basal-like breast cancer patients. Cancer Chemother Pharmacol.

[12] Ono M, Tsuda H, Shimizu C, Yamamoto S, Shibata T, Yamamoto H (2012). Tumor-infiltrating lymphocytes are correlated with response to neoadjuvant chemotherapy in triple-negative breast cancer. Breast Cancer Res Treat.

[13] Mayer IA, Abramson VG, Lehmann BD, Pietenpol JA (2014). New strategies for triple-negative breast cancer—deciphering the heterogeneity. Clin Cancer Res.

[14] Kurebayashi J, Yamamoto Y, Kurosumi M, Okubo S, Nomura T, Tanaka K (2006). Loss of BRCA1 expression may predict shorter time-to-progression in metastatic breast cancer patients treated with taxanes. Anticancer Res.

[15] Akashi-Tanaka S, Watanabe C, Takamaru T, Kuwayama T, Ikeda M, Ohyama H (2015). BRCAness predicts resistance to taxane-containing regimens in triple negative breast cancer during neoadjuvant chemotherapy. Clin Breast Cancer.

[16] Ishikawa T, Narui K, Tanabe M, Kida K, Oba MS, Yamada A (2016). BRCAness is beneficial for indicating triple negative breast cancer patients resistant to taxane. Eur J Surg Oncol.

[17] Kim MC, Choi JE, Lee SJ, Bae YK (2016). Coexistent loss of the expressions of BRCA1 and p53 predicts poor prognosis in triple-negative breast cancer. Ann Surg Oncol.

[18] Yang Q, Sakurai T, Mori I, Yoshimura G, Nakamura M, Nakamura Y (2001). Prognostic significance of BRCA1 expression in Japanese sporadic breast carcinomas. Cancer.

[19] Tanaka K, Tokunaga E, Inoue Y, Yamashita N, Saeki H, Okano S (2016). Impact of expression of vimentin and axl in breast cancer. Clin Breast Cancer.

[20] Yamashita N, Tokunaga E, Kitao H, Hisamatsu Y, Taketani K, Akiyoshi S (2013). Vimentin as a poor prognostic factor for triple-negative breast cancer. J Cancer Res Clin Oncol.

[21] Aso T, Matsuo M, Kiyohara H, Taguchi K, Rikimaru F, Shimokawa M (2015). Induction of CD44 variant 9-expressing cancer stem cells might attenuate the efficacy of chemoradioselection and Worsens the prognosis of patients with advanced head and neck cancer. PLoS One.

[22] Hirata K, Suzuki H, Imaeda H, Matsuzaki J, Tsugawa H, Nagano O (2013). CD44 variant 9 expression in primary early gastric cancer as a predictive marker for recurrence. Br J Cancer.

[23] Ishimoto T, Nagano O, Yae T, Tamada M, Motohara T, Oshima H (2011). CD44 variant regulates redox status in cancer cells by stabilizing the xCT subunit of system xc() and thereby promotes tumor growth. Cancer Cell.

[24] Hagiwara M, Kikuchi E, Kosaka T, Mikami S, Saya H, Oya M (2016). Variant isoforms of CD44 expression in upper tract urothelial cancer as a predictive marker for recurrence and mortality. Urol Oncol.

[25] Acs B, Zambo V, Vizkeleti L, Szasz AM, Madaras L, Szentmartoni G (2017). Ki-67 as a controversial predictive and prognostic marker in breast cancer patients treated with neoadjuvant chemotherapy. Diagn Pathol.

[26] Kurosumi M, Akashi-Tanaka S, Akiyama F, Komoike Y, Mukai H, Nakamura S (2008). Histopathological criteria for assessment of therapeutic response in breast cancer (2007 version). Breast Cancer.

[27] Symmans WF, Wei C, Gould R, Yu X, Zhang Y, Liu M (2017). Long-term prognostic risk after neoadjuvant chemotherapy associated with residual cancer burden and breast cancer Subtype. J Clin Oncol.

[28] Tokuda E, Horimoto Y, Arakawa A, Himuro T, Senuma K, Nakai K (2017). Differences in Ki67 expressions between pre- and post-neoadjuvant chemotherapy specimens might predict early recurrence of breast cancer. Hum Pathol.

[29] Diaz J, Stead L, Shapiro N, Newell R, Loudig O, Lo Y (2013). Mitotic counts in breast cancer after neoadjuvant systemic chemotherapy and development of metastatic disease. Breast Cancer Res Treat.

[30] Masuda N, Lee SJ, Ohtani S, Im YH, Lee ES, Yokota I (2017). Adjuvant capecitabine for breast cancer after preoperative chemotherapy. N Engl J Med.

[31] Masuda H, Baggerly KA, Wang Y, Zhang Y, Gonzalez-Angulo AM, Meric-Bernstam F (2013). Differential response to neoadjuvant chemotherapy among 7 triple-negative breast cancer molecular subtypes. Clin Cancer Res.

[32] Takayama T, Kubo T, Morikawa A, Morita T, Nagano O, Saya H (2016). Potential of sulfasalazine as a therapeutic sensitizer for CD44 splice variant 9-positive urogenital cancer. Med Oncol.

[33] Shitara K, Doi T, Nagano O, Imamura CK, Ozeki T, Ishii Y (2017). Dose-escalation study for the targeting of CD44v + cancer stem cells by sulfasalazine in patients with advanced gastric cancer (EPOC1205). Gastric Cancer.

